# A Predictive Dual-Stage Neural Framework for Phase-Coherent Auditory Synthesis on Edge Devices

**DOI:** 10.3390/s26113344

**Published:** 2026-05-25

**Authors:** Sathit Pairoch, Pattarapong Phasukkit, Teeraporn Suteewong

**Affiliations:** School of Engineering, King Mongkut’s Institute of Technology Ladkrabang, Bangkok 10520, Thailand; sathit.pa@kmitl.ac.th (S.P.); teeraporn.su@kmitl.ac.th (T.S.)

**Keywords:** auditory neuro-stimulation, binaural beats, phase-coherent synthesis, predictive signal processing, edge AI, real-time processing

## Abstract

Real-time binaural beat synthesis in dynamic acoustic environments is challenged by carrier non-stationarity, interaural phase discontinuities, and processing delay in conventional digital signal processing pipelines. This study proposes a predictive dual-stage neural framework for phase-coherent auditory synthesis under non-stationary acoustic conditions. The framework decouples real-time carrier estimation from phase-coherent signal generation through two specialized modules. An intelligent acoustic sensing module (AI-1) estimates time-varying carrier information across harmonic, fluctuating, and broadband acoustic profiles using a causal neural front-end with an adaptive confidence-driven strategy. A predictive phase-coherent generator (AI-2) then forecasts short-horizon carrier trajectories and drives a discrete-time phase accumulator to maintain continuous phase evolution during binaural beat embedding. Objective evaluation under multiple acoustic profiles and noise conditions shows that the proposed framework maintains strong phase continuity, with a Phase Coherence Factor greater than 0.91, and low artifact levels, with a Signal-to-Artifact Ratio greater than 39.8 dB, under the evaluated conditions. Additional comparisons with conventional DSP baselines, stronger classical F0 estimators, a lightweight neural F0 tracker, and component-wise ablation variants further demonstrate that the performance improvement arises from the combination of adaptive carrier estimation and predictive phase-coherent actuation, rather than from carrier estimation alone. Hardware profiling shows a combined INT8 inference time of 2.4 ms per frame on a resource-constrained Raspberry Pi Zero 2W-class edge device. Importantly, this inference time and the sub-millisecond phase-accumulator resolution should not be interpreted as sub-millisecond end-to-end physical audio latency. The complete system still includes buffering, framing, neural inference, and output processing delay; the proposed method instead reduces effective phase-boundary misalignment through short-horizon predictive compensation. These results support the proposed framework as a lightweight engineering solution for real-time phase-continuous auditory synthesis in dynamic listening environments. The reported PCF and SAR values should be interpreted as signal-level indicators of phase continuity and artifact suppression, rather than as evidence of listener comfort, perceptual preference, or neurophysiological efficacy.

## 1. Introduction

Binaural beats (BBs) represent an auditory illusion perceived when two pure tones with slightly different frequencies are presented dichotically, one to each ear. The human auditory system integrates these stimuli, resulting in the perception of a beat component whose frequency is determined by the difference between the left and right stimulus frequencies. The binaural beat frequency, denoted as $f_{beat}$, is defined as $f_{beat}=|f_{L}-f_{R}|$. Typically, $f_{beat}\;$ is selected within a low-frequency range that overlaps with major electroencephalographic frequency bands [1,2]. Although binaural beats have been investigated in relation to auditory steady-state responses, frequency-following responses, and potential brainwave entrainment [1,3,4], the present study does not aim to establish perceptual or neurophysiological efficacy. Instead, this work focuses on the engineering problem of generating phase-continuous binaural beat stimuli under dynamic and non-stationary acoustic carrier conditions.

Traditional binaural beat generation commonly relies on a static-carrier paradigm, in which a fixed carrier frequency, such as 240 Hz or 255 Hz, is maintained throughout an auditory session. However, fixed pure-tone stimuli can be perceived as monotonous during prolonged listening, which may reduce user acceptance over time [3]. Embedding binaural beats into naturalistic, musical, or dynamically varying audio may improve the practical usability of binaural beat delivery [5]. Nevertheless, integrating precise binaural beat stimuli into non-stationary acoustic carriers introduces specific signal-processing challenges, particularly because the carrier frequency may vary over time and the left–right phase relationship must remain continuous.

The fundamental engineering challenge lies in simultaneously maintaining carrier-tracking accuracy, low effective update delay, and interaural phase continuity during dynamic signal synthesis. Conventional frame-based digital signal processing approaches, such as overlapping filter banks and short-time Fourier transform (STFT)-based tracking, inherently involve a trade-off between time resolution and frequency resolution. Shorter windows improve temporal responsiveness but reduce frequency precision, whereas longer windows improve spectral precision at the cost of increased delay. In binaural synthesis, such delay or frame-boundary mismatch can disturb the intended interaural phase relationship and generate audible artifacts, clicks, or unintended beating patterns [6,7,8,9].

Classical phase-tracking methods, such as phase-locked loops (PLLs), provide continuous tracking under quasi-stationary conditions, but may suffer from lock-in delay, overshoot, or transient tracking errors when the acoustic carrier changes rapidly [7,8,9]. These limitations become more critical when binaural beat signals are embedded into non-stationary or broadband soundscapes, where the dominant carrier may become ambiguous, fluctuate across time, or disappear temporarily. Therefore, dynamic binaural beat synthesis requires more than current-frame carrier estimation; it also requires a mechanism that anticipates short-horizon carrier variation and updates the synthesized phase trajectory smoothly.

Recent progress in artificial intelligence has significantly improved acoustic sensing, pitch estimation, sound event detection, audio restoration, and neural audio synthesis [10,11,12,13]. However, many AI-based acoustic perception and restoration models operate primarily on magnitude-domain representations, such as Mel-spectrograms, amplitude spectra, or time–frequency magnitude masks. These models are usually optimized for classification, denoising, source separation, enhancement, or perceptual reconstruction. Although such approaches can be highly effective for recognition and restoration tasks, they do not directly impose deterministic future-oriented interaural phase constraints. In many cases, phase is either reconstructed implicitly, treated as a secondary variable, or estimated after magnitude-domain processing.

This distinction is important for dynamic binaural beat synthesis. The objective is not only to estimate the present carrier frequency, but also to preserve a continuous relationship between the left and right channel phases while the carrier changes over time. Any delay, discontinuous frame update, or unstable phase correction can perturb the intended binaural beat frequency and introduce signal artifacts. Therefore, a model that performs well in magnitude reconstruction, pitch classification, or acoustic scene analysis may still fail to guarantee phase-continuous binaural beat embedding under real-time non-stationary conditions.

To address this gap, this study formulates dynamic binaural beat synthesis as a predictive phase-coherence control problem rather than as a conventional magnitude-domain audio reconstruction problem. The proposed framework decouples the task into two specialized stages. The first stage, referred to as the intelligent acoustic sensing module (AI-1), estimates the time-varying carrier trajectory from non-stationary acoustic inputs. The second stage, referred to as the predictive phase-coherent generator (AI-2), forecasts the short-horizon carrier trajectory and updates a discrete-time phase accumulator to reduce effective phase-boundary misalignment during synthesis. This formulation treats phase as a dynamic state variable rather than a post hoc reconstruction output.

The innovation of this work does not lie in introducing a new neural network layer, but in the system-level and methodological integration of adaptive carrier sensing, predictive phase compensation, and deterministic phase-continuous actuation. Existing STFT-based methods estimate carrier information from frame-wise spectra but can suffer from frame-boundary discontinuities. PLL-based methods provide continuous tracking under limited conditions but may respond slowly to rapid carrier variation. Magnitude-spectrum-based AI models can classify or reconstruct audio features but generally do not enforce future-oriented interaural phase continuity. Generic neural audio generators can synthesize waveforms but are not specifically constrained to preserve a target binaural beat-frequency relationship under real-time carrier variation. In contrast, the proposed framework separates carrier estimation from phase-coherent actuation and constrains the final waveform generation through an explicit phase accumulator.

It is important to clarify the boundary of the performance claims in this study. The proposed framework does not eliminate physical buffering, framing, neural inference, or audio output delay. Instead, it aims to reduce the effect of such delay on phase-continuous synthesis by predicting short-horizon carrier trajectories and updating the phase accumulator accordingly. Thus, any discussion of low effective delay or high phase resolution in this work refers to effective phase-boundary alignment and phase-accumulator computation under the evaluated conditions, not to sub-millisecond end-to-end physical audio latency.

The specific contributions of this paper are as follows. First, we propose a predictive dual-stage neural/DSP framework for dynamic binaural beat synthesis under non-stationary acoustic conditions. Second, we develop an adaptive carrier-estimation module that combines causal neural feature extraction with confidence-driven frequency tracking across tonal, harmonic/fluctuating, and broadband acoustic profiles. Third, we introduce a predictive phase-coherent generator that forecasts short-horizon carrier trajectories and drives a discrete-time phase accumulator to preserve continuous phase evolution. Fourth, we evaluate the framework using objective engineering metrics, including frequency tracking error, Phase Coherence Factor (PCF), Signal-to-Artifact Ratio (SAR), tracking latency, and edge-device execution time. Finally, we compare the proposed system with conventional DSP baselines, stronger classical pitch estimators, a lightweight neural F0 baseline, and component-wise ablation variants to clarify the contribution of each module.

The present study should be interpreted as a signal-level and systems-level engineering evaluation. The reported PCF and SAR values quantify mathematical phase continuity and artifact suppression under benchmark conditions, but they do not directly establish listener comfort, perceptual preference, or neurophysiological efficacy. Controlled listening experiments and physiological validation using electroencephalography, auditory steady-state response, or frequency-following response measurements remain necessary future work before any claims regarding perceptual or neurophysiological benefit can be made.

The remainder of this paper is organized as follows. Section 2 presents the mathematical framework and reviews related work on phase-coherent binaural beat synthesis, conventional DSP limitations, and AI-based acoustic sensing. Section 3 describes the proposed dual-stage architecture, including AI-1 for adaptive carrier estimation and AI-2 for predictive phase-coherent generation. Section 4 details the experimental design, acoustic profiles, baseline systems, evaluation metrics, and edge-hardware profiling. Section 5 presents the experimental results, including baseline comparisons, ablation studies, and signal-level performance analysis. Section 6 discusses the engineering implications, trade-offs, limitations, and future validation requirements. Finally, Section 7 concludes the paper.

## 2. Mathematical Framework and Related Work

### 2.1. Formulating the Phase-Coherent Binaural Beat

To embed a stable binaural beat within a non-stationary acoustic carrier, it is necessary to control the interaural phase relationship directly rather than only updating the left and right channel frequencies independently. Because instantaneous frequency is the time derivative of phase, the dynamic binaural beat frequency can be expressed as

(1) $$f_{beat}(t)=\frac{1}{2\pi }\left|\frac{{d\Phi }_{L}(t)}{dt}-\frac{{d\Phi }_{R}(t)}{dt}\right|$$

where $\Phi_{L}\left(t\right)\;$ and $\Phi_{R}\left(t\right)\;$ denote the instantaneous phases of the left and right acoustic channels, respectively. To maintain a constant target binaural beat frequency, $f_{beat}$, while the background carrier varies over time, the phase difference between the two channels should satisfy

(2) $$\left|\Phi_{L}(t)-\Phi_{R}(t)\right|=2\pi f_{beat}t+{∆\phi }_{0}$$

where $\Delta \phi_{0}\;$ represents the initial interaural phase offset. This relationship indicates that binaural beat synthesis under a time-varying carrier is fundamentally a phase-control problem rather than only a frequency-estimation problem [1,2,14,15,16,17,18,19,20].

Under a symmetric binaural embedding strategy, the left and right stimulus frequencies are defined relative to the time-varying carrier frequency, $f_{carrier}(t)$, as

(3) $$f_{L}(t)=f_{carrier}(t)+\frac{f_{beat}}{2}$$

(4) $$f_{R}(t)=f_{carrier}(t)-\frac{f_{beat}}{2}$$

Thus, both channels must track the same time-varying carrier while preserving the target interaural frequency offset. However, if these frequencies are updated in a discontinuous frame-wise manner, the resulting phases may not remain continuous across update boundaries. To avoid this problem, the left and right channel phases should be obtained by integrating the instantaneous frequencies over time:

(5) $$\Phi_{L}(t)=2\pi \int_{0}^{t}\left[f_{carrier}(\tau )+\frac{f_{beat}}{2}\right]d\tau +\Phi_{L,0}$$

(6) $$\Phi_{R}(t)=2\pi \int_{0}^{t}\left[f_{carrier}\left(\tau \right)-\frac{f_{beat}}{2}\right]d\tau +\Phi_{R,0}$$

where $\Phi_{L,0\;}$ and $\Phi_{R,0}\;$ denote the initial phases of the left and right channels, respectively. The synthesized dichotic signals are then represented as

(7) $$S_{L}(t)=A_{L}(t)sin(\Phi_{L}(t))$$

(8) $$S_{R}(t)=A_{R}(t)sin(\Phi_{R}(t))$$

where $A_{L}\left(t\right)\;$ and $A_{R}\left(t\right)\;$ denote the time-varying amplitude envelopes of the left and right channels.

This formulation relies on several assumptions. First, the background audio should contain either a dominant carrier frequency or a meaningful spectral descriptor that can be tracked over time. Second, the carrier-frequency estimate should be sufficiently stable for phase integration. Third, the amplitude envelopes $A_{L}\left(t\right)\;$ and $A_{R}\left(t\right)\;$ should vary slowly relative to the carrier so that envelope modulation does not introduce unintended phase distortion. This assumption is consistent with the spectral-separation condition described by Bedrosian’s theorem for Hilbert-transform product separation [21]. Under these conditions, any carrier-tracking delay, frame-boundary mismatch, or discontinuous frequency update can induce phase mismatch and generate audible artifacts [8,20].

### 2.2. Limitations of Legacy Signal Processing

Conventional digital signal processing methods provide useful foundations for carrier estimation, but they face structural limitations in non-stationary acoustic environments. The short-time Fourier transform (STFT), for example, estimates spectral content using a sliding analysis window. This approach is constrained by the time–frequency resolution trade-off: shorter windows improve temporal responsiveness but reduce frequency precision, whereas longer windows improve spectral resolution but introduce larger analysis delay [22,23]. For dynamic binaural beat synthesis, this trade-off is important because frequency estimates must be updated rapidly while maintaining phase-continuous output.

STFT-based peak tracking can estimate a dominant spectral component from frame-wise magnitude spectra, but the resulting oscillator updates are often tied to analysis-frame boundaries. When the carrier changes rapidly, frame-wise updates can introduce discontinuities or phase-boundary mismatch. Although overlap-add processing can reduce some audible artifacts in general audio processing, it does not by itself guarantee deterministic interaural phase continuity for binaural beat embedding. Therefore, STFT-based methods remain limited when strict phase-continuous synthesis is required under non-stationary carrier conditions [22,23].

Phase-locked loops (PLLs) provide another classical approach for continuous frequency and phase tracking. They can perform well under quasi-stationary or slowly varying conditions, but their tracking behavior depends on loop-filter settings and lock-in dynamics. In rapidly changing acoustic scenes, PLL-based approaches may exhibit response delay, overshoot, or transient tracking error [24,25,26]. Increasing the smoothing or filtering strength can improve noise robustness, but this also increases latency. Thus, PLL-based approaches also face a trade-off between tracking stability and responsiveness [24,25,26].

These limitations do not imply that STFT or PLL methods are unsuitable in general. Rather, they indicate that conventional DSP approaches are not specifically designed for the combined requirements of dynamic binaural beat synthesis: robust carrier estimation, low effective update delay, and deterministic interaural phase continuity. This motivates a framework in which carrier estimation and phase-continuous synthesis are treated as related but distinct system objectives.

### 2.3. AI in Acoustic Sensing and Synthesis: The Predictive Phase-Coherence Gap

Deep learning has substantially improved acoustic sensing, pitch estimation, audio enhancement, sound event detection, source separation, and neural synthesis [27,28,29,30,31]. Many of these approaches use magnitude-domain representations, such as Mel-spectrograms, amplitude spectra, or time–frequency magnitude masks. These representations are effective for classification, denoising, restoration, and perceptual reconstruction because they capture salient spectral and temporal patterns. However, they are not inherently designed to enforce deterministic future-oriented phase continuity between two audio channels [29,30,31].

This distinction is critical for dynamic binaural beat synthesis. In many acoustic perception or restoration tasks, phase can be reconstructed implicitly, estimated post hoc, or treated as a secondary variable after magnitude-domain processing. In contrast, binaural beat synthesis requires the left and right channel phases to evolve continuously while preserving a target interaural frequency difference. A model that accurately classifies an acoustic scene or reconstructs a magnitude spectrum may still produce discontinuous phase updates if it is not explicitly constrained by a phase-state model. Therefore, current magnitude-spectrum-based AI models cannot be directly applied to the strict interaural phase-continuity requirement of this task [29,30,31].

Existing neural audio generators also differ from the present objective. Generic waveform generators, vocoders, and restoration networks can synthesize high-quality audio, but they are usually optimized for perceptual waveform quality, spectral reconstruction, or distributional similarity. They are not specifically designed to maintain a deterministic binaural beat-frequency relationship under real-time carrier variation. In addition, end-to-end neural waveform synthesis may provide limited interpretability when a phase error occurs, because carrier estimation, prediction, and waveform actuation are entangled within a single model [29,30,31].

The critical research gap is therefore not simply the lack of another neural pitch detector or waveform generator. Rather, the gap lies in predictive phase-coherent control: the system must estimate the current carrier, forecast its short-horizon trajectory, and update the left and right channel phases in a constrained manner. Without a predictive and phase-aware objective, even an accurate carrier estimator can still produce effective phase-boundary mismatch when its output is used by a non-predictive generator.

To address this gap, the present work formulates dynamic binaural beat synthesis as a predictive phase-state control problem. The proposed AI-1 module estimates the time-varying carrier trajectory from non-stationary acoustic input, while the AI-2 module forecasts the short-horizon carrier state and updates a discrete-time phase accumulator. This design treats phase as a dynamic state variable and constrains waveform generation through explicit phase accumulation rather than unconstrained frame-wise synthesis. The purpose is not to eliminate physical buffering or processing delay, but to reduce the effect of such delay on phase-boundary alignment during real-time synthesis.

### 2.4. Comparative Positioning and Innovation Boundary

The proposed framework differs from existing approaches in both objective and constraint structure. Conventional STFT-based methods focus on frame-wise spectral estimation, but they may introduce phase-boundary discontinuities when the carrier is updated across frames [22,23]. PLL-based methods provide continuous tracking under limited conditions, but they can suffer from lock-in delay and reduced responsiveness during rapid frequency transitions [24,25,26]. Magnitude-spectrum-based AI acoustic models are effective for classification, enhancement, denoising, and restoration, but they generally do not impose future-oriented interaural phase constraints [27,28,29,30,31]. Generic neural audio generators can synthesize waveforms, but they are not designed to preserve a target binaural beat-frequency relationship under real-time carrier variation [29,30,31]. A comparison between the proposed framework and related approaches is summarized in Table 1.

The innovation of this work therefore lies in the formulation and integration of a predictive phase-coherent synthesis pipeline rather than in the invention of a new neural network layer. AI-1 and AI-2 are constructed using established neural building blocks, but they are assigned different control objectives. AI-1 focuses on robust carrier estimation, whereas AI-2 focuses on short-horizon prediction, phase-continuous actuation, and reduction of effective phase-boundary misalignment. This separation improves interpretability, supports modular updating, and constrains the final waveform through a deterministic phase accumulator.

Accordingly, the proposed framework should be interpreted as a signal-level and systems-level engineering solution for phase-continuous dynamic auditory synthesis. It is not intended to provide direct evidence of listener comfort, perceptual preference, or neurophysiological efficacy. Those questions require controlled human auditory experiments and physiological validation, which are beyond the scope of the present benchmark and are identified as future work.

## 3. Proposed Dual-AI Architecture

To overcome the latency, tracking, and phase-boundary limitations of conventional frame-based digital signal processing pipelines, this study proposes a predictive Dual-AI architecture for dynamic binaural beat synthesis. The system separates the real-time processing pipeline into two specialized modules: an intelligent acoustic sensing module (AI-1) and a predictive phase-coherent generator (AI-2). AI-1 estimates the time-varying carrier information from non-stationary acoustic inputs, whereas AI-2 forecasts the short-horizon carrier trajectory and drives a constrained phase accumulator to preserve continuous phase evolution during binaural beat embedding.

The rationale for this decoupled design is that carrier estimation and phase-continuous synthesis are related but distinct system objectives. AI-1 is optimized primarily for robust carrier tracking under tonal, harmonic/fluctuating, and broadband acoustic conditions. In contrast, AI-2 is optimized for predictive smoothness and phase-boundary continuity during real-time synthesis. This separation improves interpretability because carrier-estimation errors and phase-generation errors can be analyzed independently. It also supports modular updating, since the sensing and generation stages can be improved separately without redesigning the entire system.

**Figure 1:**
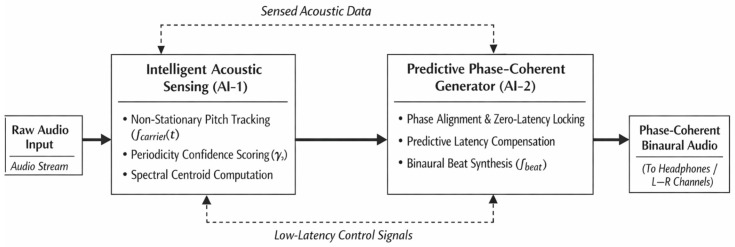
Proposed Dual-AI architecture, decoupling real-time intelligent acoustic sensing (AI-1) from predictive phase-coherent synthesis (AI-2).

Importantly, the proposed architecture does not eliminate physical buffering, framing, neural inference, or audio output delay. Instead, it aims to reduce the effect of such delay on the synthesized waveform by forecasting short-horizon carrier variation before updating the phase accumulator (Figure 1). Therefore, terms such as low effective delay or phase-boundary alignment in this study refer to reduced effective phase mismatch under the evaluated conditions, not to physical zero-latency audio delivery.

### 3.1. Intelligent Acoustic Sensing Module (AI-1): Real-Time Non-Stationary Pitch Tracker

The primary objective of AI-1 is to estimate the instantaneous background carrier frequency, $f_{carrier}(t)$, from non-stationary acoustic inputs. Operating as a lightweight auditory front-end, AI-1 combines causal one-dimensional convolutional neural network (1D-CNN) layers with a uni-directional LSTM and a temporal attention mechanism. This design enables real-time carrier estimation while avoiding future-frame leakage, which is important for causal edge-oriented implementation.

#### 3.1.1. Network Architecture and Feature Extraction

The AI-1 module receives raw audio waveforms downsampled to 16 kHz. To balance temporal responsiveness and spectral information, the audio stream is segmented into 64 ms sliding windows, corresponding to 1024 samples, with a 10 ms hop size. This configuration provides a 100 Hz algorithmic update rate. The total parameter count of AI-1 is approximately 285 K parameters.

The AI-1 architecture consists of three main sub-components. First, a causal 1D-CNN pitch encoder extracts hierarchical temporal features from the input waveform. The encoder uses three causal convolutional layers with kernel sizes k={16,8,4}, stride 2, and channel dimensions c={32,64,128}. Batch normalization and Mish activation functions are applied after the convolutional layers to improve convergence and nonlinear feature representation.

Second, a single-layer uni-directional LSTM with a hidden size of h=128 models temporal dependencies in the extracted feature sequence. A temporal attention mechanism is then applied to weight the LSTM hidden states. This mechanism emphasizes frames with stronger harmonic or periodic evidence while reducing the influence of transient disturbances and noisy frames.

Third, the attended latent representation is passed to two parallel output heads. The Pitch Head outputs a pseudo-probability distribution over 360 quantized frequency bins covering the range from 50 Hz to 1000 Hz. The Confidence Head outputs a scalar periodicity score, $\gamma_{t}\in [0,\;1]$, which estimates the harmonic stability of the current frame. This confidence score is used by the adaptive dual-path extraction strategy described in Section 3.1.2.

**Figure 2:**
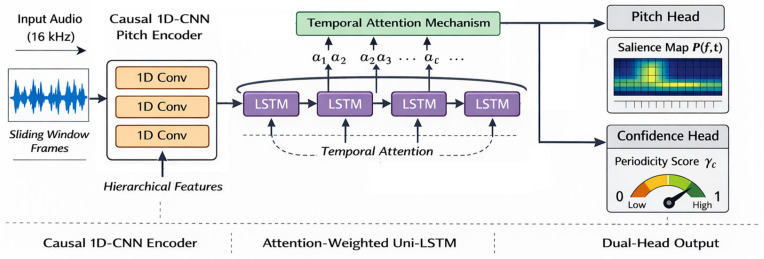
Architecture of the AI-1 module, using a causal 1D-CNN and attention-weighted LSTM for dual-head carrier-frequency tracking in non-stationary acoustic environments.

The architecture of the AI-1 module is illustrated in Figure 2.

#### 3.1.2. Adaptive Dual-Path Frequency Extraction

To support carrier tracking across different acoustic profiles, AI-1 uses an adaptive dual-path extraction strategy. If the periodicity confidence score exceeds a predefined threshold,

$$\left(\gamma_{t}\geq \tau \right)$$

the input frame is treated as harmonic or tonal. In this case, the dominant carrier estimate is obtained from the Pitch Head by selecting the frequency bin with the maximum posterior probability:

$$f_{t}^{*}=arg{max}_{f}P(f,t)$$

A Viterbi-based smoothing step is then applied to reduce non-physical octave jumps and stabilize the estimated carrier trajectory [32,33,34,35,36,37,38,39,40,41].

If the periodicity confidence is lower than the threshold,

$$\left(\gamma_{t}\geq \tau \right)$$

the frame is treated as broadband or non-pitched. In such cases, estimating a fundamental pitch is not well defined. Therefore, the system computes the spectral centroid from the magnitude spectrum as a meaningful broadband carrier descriptor [42,43,44,45]:

$$C(t)=\frac{\sum_{k}f_{k}|X_{t}(k)|}{\sum_{k}|X_{t}(k)|}$$

where $X_{t}(k)$ denotes the magnitude spectrum at frequency bin k, and $f_{k}$ is the corresponding frequency. The resulting carrier estimate is then forwarded to AI-2 for predictive phase-coherent synthesis.

**Figure 3:**
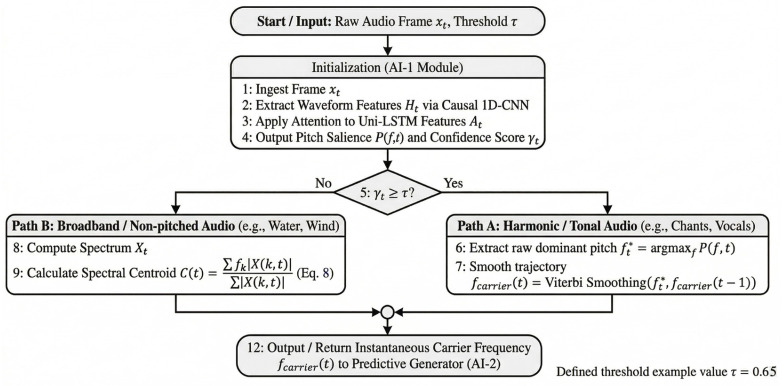
Adaptive dual-path extraction in AI-1. A confidence-driven gate, $\gamma_{t}$, switches between harmonic pitch tracking and broadband spectral-centroid tracking to provide a continuous carrier descriptor for AI-2.

The adaptive dual-path extraction strategy used in AI-1 is summarized in Figure 3.

### 3.2. Predictive Phase-Coherent Generator Module (AI-2)

Although AI-1 provides a carrier estimate, the complete real-time system still contains buffering, framing, neural inference, and output processing delay. If the estimated carrier is applied directly to a frame-wise oscillator, rapid carrier changes can still create effective phase-boundary mismatch. AI-2 addresses this problem by operating as a short-horizon predictor and constrained phase-actuation module.

The role of AI-2 is not to eliminate physical processing delay. Rather, AI-2 forecasts the near-future carrier trajectory and updates a discrete-time phase accumulator so that the synthesized waveform evolves smoothly across update boundaries. This design reduces the effect of algorithmic delay on phase-continuous synthesis and improves artifact suppression under non-stationary carrier conditions.

#### 3.2.1. Hybrid TCN–LSTM Forecasting

AI-2 predicts the future frequency trajectory $\hat{f}\left(t+\Delta t\right)\;$ using a historical sequence length of L=10  frames, corresponding to 100 ms of past context. The forecast horizon is set to Δt=30 ms which corresponds to the approximate algorithmic delay associated with buffering, framing, and neural inference under the evaluated implementation. The AI-2 module contains approximately 140 K parameters and uses a hybrid TCN–LSTM architecture. The Temporal Convolutional Network (TCN) consists of three dilated causal convolutional layers with dilation factors $d=\left\{1,2,4\right\}$, kernel size 3, and 64 channels. The dilated causal convolutions capture short-term localized frequency variations, such as vibrato-like changes or rapid carrier transitions. Residual connections are used to stabilize temporal feature propagation.

The TCN output is then passed to an LSTM layer with a hidden size of 64. The LSTM refinement stage models longer temporal dependencies and smooths the predicted carrier trajectory across successive frames [46,47,48,49,50,51]. A final dense layer outputs the short-horizon carrier prediction used by the phase accumulator.

**Figure 4:**
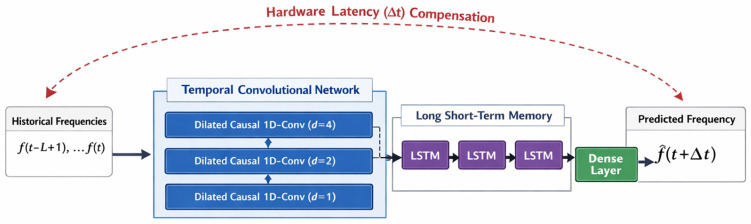
Hybrid TCN–LSTM architecture of AI-2. The module predicts the short-horizon carrier frequency, $\hat{f}(t+\Delta t)$, from historical carrier estimates to reduce effective phase-boundary misalignment caused by processing delay.

The hybrid TCN–LSTM forecasting architecture of AI-2 is shown in Figure 4.

#### 3.2.2. Phase-Locking Actuation and Boundary Constraints

To convert the predicted carrier trajectory into a continuous waveform, AI-2 uses a discrete-time phase accumulator. At a sampling rate $f_{s}\;=\;\frac{1}{Ts}$ the base phase is updated as

(9) $$\phi_{0}[n+1]=\phi_{0}[n]+2\pi \hat{f}[n]T_{s}$$

where $\phi_{0}[n]$ denotes the accumulated base phase at sample index n, $\hat{f}[n]$ denotes the predicted carrier frequency, and $T_{s}$ is the sampling interval. This recurrence ensures that phase evolves continuously from one sample to the next, rather than being reset at frame boundaries.

The target binaural beat offset is then applied symmetrically to generate the left and right channel phases:

(10) $$\phi_{L}[n+1]=\phi_{L}[n]+2\pi \left(\hat{f}[n]+\frac{f_{beat}}{2}\right)T_{s}$$

(11) $$\phi_{R}[n+1]=\phi_{R}[n]+2\pi \left(\hat{f}\left[n\right]-\frac{f_{beat}}{2}\right)T_{s}$$

The left and right output signals are synthesized as

(12) $$s_{L}[n]=A_{L}[n]sin(\phi_{L}[n])$$

(13) $$s_{R}[n]=A_{R}[n]sin(\phi_{R}[n])$$

where $A_{L}\left[n\right]\;$ and $A_{R}\left[n\right]\;$ are the time-varying amplitude envelopes of the left and right channels, respectively.

This phase-accumulator formulation prevents discontinuous phase resets during frequency updates. In the present study, the term sub-millisecond refers only to the computational resolution of phase accumulation and residual phase-boundary alignment under the evaluated conditions. It should not be interpreted as end-to-end physical latency below 1 ms. The complete system still includes physical buffering, frame construction, inference, and audio output delay.

**Figure 5:**
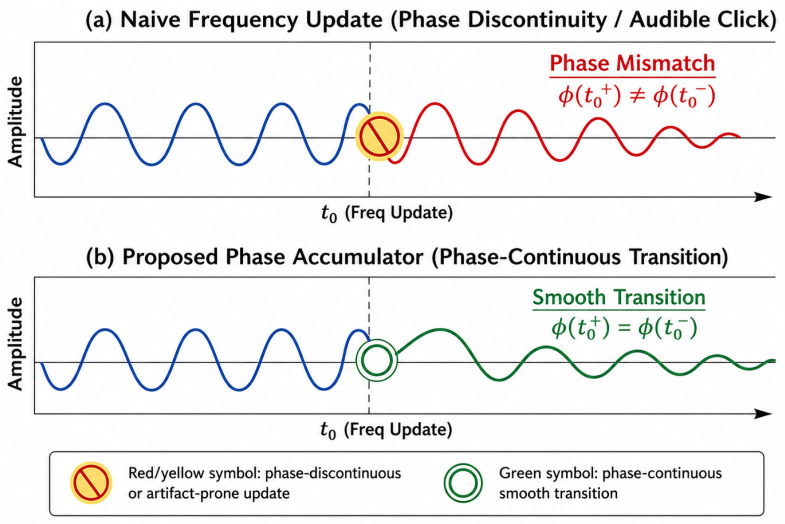
Phase-locking actuation in AI-2. (**a**) Naive frame-based frequency updates can introduce phase mismatch at transition boundaries. (**b**) The proposed predictive phase accumulator preserves continuous phase evolution and reduces effective phase-boundary misalignment. The red/yellow symbol denotes phase-discontinuous or artifact-prone frame updates, whereas the green symbol denotes phase-continuous predictive phase-accumulator-based synthesis.

The phase-locking actuation mechanism and phase-accumulator-based synthesis process are illustrated in Figure 5.

### 3.3. Training Protocol and Implementation Details

To ensure reproducibility, AI-1 and AI-2 were trained independently before system-level integration. The training dataset comprised 120 h of heterogeneous audio, augmented using dynamic signal-to-noise ratio conditions ranging from −5 dB to +20 dB. For harmonic audio samples, reference carrier-frequency labels were generated offline using a high-resolution pitch-estimation procedure applied to clean source tracks before augmentation. These reference labels were used for supervised training of the carrier-estimation module.

AI-1 was trained for 100 epochs using a 70/15/15 training, validation, and testing split. The Pitch Head was optimized using categorical cross-entropy loss, while the Confidence Head was optimized using binary cross-entropy loss. AI-2 was trained to forecast the carrier trajectory Δt=30 ms ahead of the current frame. To reduce sensitivity to transient outliers, the Huber loss function with δ=1.0  was used instead of standard mean squared error.

Both networks were optimized using the Adam optimizer with $\beta_{1}=0.9$, $\beta_{2}=0.999$, an initial learning rate of $1\times 10^{-4}$, and a batch size of 256. Early stopping was applied with a patience of 10 epochs based on validation loss to reduce overfitting.

After training, the models were quantized to INT8 precision for edge-device profiling. The deployed system uses AI-1 for real-time carrier estimation and AI-2 for predictive phase-continuous actuation. The objective of the hardware implementation is to evaluate lightweight real-time feasibility on a resource-constrained edge platform, rather than to demonstrate wearable-scale ultra-low-power operation or physical zero-latency audio delivery.

### 3.4. Boundary Conditions of the Proposed Architecture

The proposed architecture should be interpreted within the engineering scope of this study. First, the framework is designed to improve signal-level phase continuity and reduce artifact energy during dynamic binaural beat synthesis. It is not intended to establish listener comfort, perceptual preference, or neurophysiological efficacy. Second, the system reduces effective phase-boundary misalignment through predictive compensation, but it does not remove physical buffering, framing, inference, or output latency. Third, the phase accumulator provides high computational phase resolution, but this does not imply sub-millisecond end-to-end audio latency.

Accordingly, the main contribution of the proposed Dual-AI architecture is to provide a modular and interpretable operating point for dynamic auditory synthesis in which carrier-estimation accuracy, predictive smoothness, phase-continuous actuation, and edge-device feasibility are considered jointly. Future work involving controlled listening experiments and physiological validation will be necessary to evaluate perceptual and neurophysiological utility beyond the signal-level engineering metrics considered in this study.

## 4. Experimental Design and Evaluation Methodology

This section describes the evaluation framework used to assess the proposed predictive phase-coherent binaural beat synthesis system under controlled acoustic conditions. The objective of the evaluation is to compare the proposed method against representative conventional signal-processing baselines, stronger classical F0 estimators, a lightweight neural F0 estimation baseline, and ablation variants of the proposed framework. The evaluation focuses on carrier-tracking accuracy, phase continuity, response behavior, artifact suppression, and lightweight edge-device feasibility. The study is intentionally framed as a signal-level and systems-level engineering evaluation rather than as a perceptual or neurophysiological validation study.

### 4.1. Dataset and Acoustic Profiles

In this study, non-stationarity is operationally defined as short-time variation in one or more acoustic properties that directly affect binaural beat embedding, including carrier-frequency trajectory, harmonic periodicity, spectral bandwidth, and noise level. The targeted operating range is therefore not limited to a fixed carrier tone, but includes acoustic scenes in which the dominant carrier changes over time, becomes partially ambiguous due to overlapping partials, or becomes unavailable under broadband or noisy conditions. This definition aligns the experimental benchmark with the phase-coherence problem formulated in Section 2, where carrier-estimation error, frame-boundary updates, and algorithmic delay can lead to interaural phase discontinuities.

To evaluate robustness across representative dynamic listening conditions, the test audio was organized into three acoustic profiles and standardized to a sampling rate of 16 kHz. The first profile, Tonal/Melodic, includes signals with clear harmonic structure and well-defined fundamental frequencies, such as monophonic instruments, sustained notes, or solo vocal content. This profile evaluates high-precision carrier tracking in structured but time-varying acoustic environments.

The second profile, Harmonic/Fluctuating, includes more complex but still partially periodic material, such as vocal drones, chants, or slowly varying harmonic textures. This profile is intended to test robustness under overlapping partials and moderate temporal instability. The third profile, Noisy/Broadband, includes signals without a stable fundamental frequency, such as flowing water, ambient soundscapes, or diffuse acoustic textures. In this case, the evaluation specifically tests the broadband fallback mechanism described in Algorithm 1.
**Algorithm 1:** Adaptive Dual-Path Frequency Extraction for Real-Time Acoustic SensingInput: Raw audio frame $x_{t}$, confidence threshold τ Output: Instantaneous carrier estimate $f_{carrier}(t)$ 1: Ingest the raw audio frame $x_{t}$ into AI-1. 2: Extract latent waveform features:            Ht←Causal_1D−CNN(xt) 3: Apply attention-weighted temporal modeling:          At←Attention−Weighted LSTM(Ht) 4: Estimate pitch distribution and periodicity confidence:          $P(f,t),\gamma_{t}\leftarrow Dual-Head\;Network(At)$ 5: If $\gamma_{t}\geq \tau$, use the harmonic path:              $f_{t}^{*}=arg{max}_{f}P(f,t)$         $f_{carrier}(t)\leftarrow Viterbi\_Smoothing(f_{t}^{*},f_{carrier}(t-1))$ 6: Otherwise, use the broadband path:        Xt←Magnitude_Spectrum(xt)           $f_{carrier}(t)\leftarrow \frac{\sum_{k}f_{k}|X_{t}(k)|}{\sum_{k}|X_{t}(k)|}$ 7: Return $f_{carrier}(t)$ to the predictive phase-coherent generator (AI-2).

Accordingly, the three acoustic profiles were selected to span representative levels of non-stationarity within the scope of this study. The Tonal/Melodic profile represents structured but time-varying carrier trajectories; the Harmonic/Fluctuating profile represents more complex partial structures with moderate temporal instability; and the Noisy/Broadband profile represents diffuse acoustic scenes in which a stable fundamental frequency is absent and the centroid-based fallback pathway is required.

**Figure 6:**
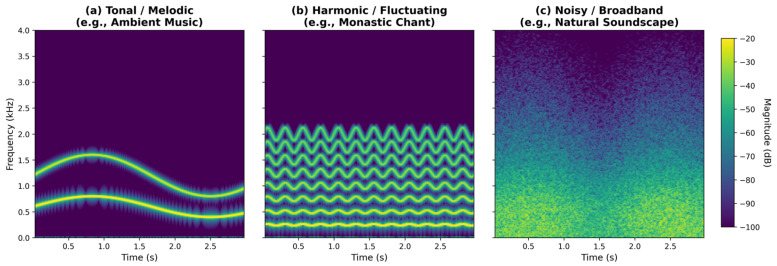
Representative spectrograms of the three acoustic profiles used for evaluation: (**a**) Tonal/Melodic with stable harmonic contours, (**b**) Harmonic/Fluctuating with denser and less stable partial structure, and (**c**) Noisy/Broadband with diffuse spectral energy.

To assess noise robustness, each acoustic profile was evaluated under four signal-to-noise ratio (SNR) conditions: Clean, 20 dB, 10 dB, and 0 dB. This design provides a common benchmark for comparing algorithm behavior across both favorable and degraded signal conditions. In addition, abrupt carrier-transition tests, such as 220 Hz to 440 Hz, were used in the latency evaluation to examine response behavior under rapid frequency change. Representative spectrograms of the three acoustic profiles are shown in Figure 6.

### 4.2. Baseline Systems for Comparison

The proposed framework was evaluated against several comparison systems designed to represent conventional DSP baselines, stronger classical F0 estimators, lightweight neural F0 estimation, and ablation variants of the proposed architecture. All methods were evaluated under the same acoustic profiles, SNR conditions, sampling rate, frame configuration, and objective metrics.

**B1: STFT-VFO (Short-Time Fourier Transform Variable Frequency Oscillator).**

This baseline estimates the carrier frequency from the peak of the STFT magnitude spectrum and updates a sinusoidal oscillator on a frame-by-frame basis. It provides a conventional frame-based reference that is expected to be affected by the time–frequency resolution trade-off and by phase discontinuities at frame boundaries.

**B2: DSOGI-PLL (Dual Second-Order Generalized Integrator Phase-Locked Loop).** This baseline represents a classical time-domain phase-tracking approach. The proportional-integral controllers were tuned using the Ziegler–Nichols method and configured for a center-frequency operating range of 50 Hz to 1000 Hz, covering the principal range of interest for the present auditory carrier-tracking setting.

**B3: Ablation Model (AI-1 + Naive Generator).**

This configuration combines the proposed AI-1 carrier-estimation front-end with a basic non-predictive sinusoidal generator. It is included to isolate the contribution of AI-2 and determine whether accurate carrier estimation alone is sufficient to achieve smooth phase-coherent synthesis.

The proposed system integrates both AI-1 and AI-2, thereby coupling adaptive carrier estimation with predictive phase-aware generation. Modern deep learning audio generation models, such as generic vocoders or WaveNet-type systems, were not included in the baseline set because they are primarily designed for waveform generation rather than low-latency deterministic phase tracking under the edge-oriented real-time setting considered in this study.

**B4: Lightweight Neural F0 Tracker.**

To compare the proposed framework with an AI-based fundamental-frequency estimation approach, a lightweight neural F0 detection baseline was implemented. This baseline uses a compact CNN-based pitch-tracking model to estimate the current carrier frequency from the same input audio stream. The estimated carrier trajectory is then passed to the same non-predictive sinusoidal generator used in the ablation configuration. This baseline is included to determine whether lightweight neural frequency estimation alone is sufficient to achieve phase-coherent binaural beat synthesis, or whether the predictive phase-coherent generator is necessary.

**B5: YIN-Based F0 Tracker.**

To strengthen the comparison with established pitch-estimation methods, a YIN-based fundamental-frequency tracking baseline was added [52]. YIN estimates the carrier trajectory using an autocorrelation-based difference function and a cumulative mean-normalized difference criterion. The estimated carrier was then passed to the same non-predictive sinusoidal generator used for the carrier-tracking baselines. This configuration evaluates whether a stronger classical F0 estimator alone is sufficient to maintain phase-coherent binaural beat synthesis under non-stationary acoustic conditions.

**B6: SWIPE-Based F0 Tracker.**

A SWIPE-based baseline was also included as another established pitch-estimation reference [53]. SWIPE estimates pitch by evaluating the similarity between the spectrum and a sawtooth-inspired harmonic template. As with the YIN baseline, the estimated carrier trajectory was used to drive the same non-predictive generator. This baseline provides a stronger comparison than simple STFT peak tracking, particularly for tonal and harmonic acoustic profiles.

**Proposed AI-1 + AI-2 Framework.**

The proposed system integrates adaptive carrier estimation with predictive phase-aware generation. AI-1 estimates the time-varying carrier trajectory, while AI-2 forecasts the short-horizon carrier state and updates the discrete-time phase accumulator. This configuration evaluates the complete predictive phase-coherent synthesis framework.

Modern generic neural audio generation models, such as general-purpose vocoders or WaveNet-type systems, were not included as direct baselines because they are primarily designed for waveform generation or reconstruction rather than low-latency deterministic phase tracking under an explicit binaural beat-frequency constraint. Instead, the added lightweight neural F0 tracker provides a more targeted.

### 4.3. Objective Evaluation Metrics

To assess performance under a common engineering benchmark, the systems were evaluated using four objective metrics. These metrics quantify carrier-tracking accuracy, phase continuity, response behavior, and signal integrity under the evaluated conditions.

In the present study, PCF and SAR are used as engineering proxy metrics for phase continuity and artifact suppression. They are not interpreted as direct measures of listener comfort, subjective preference, perceptual transparency, or neurophysiological efficacy. Formal perceptual and physiological validation therefore remains outside the scope of this hardware–software evaluation phase.

**Frequency Tracking Error (**$RMSE_{f}$**):** The frequency tracking error is calculated as the root mean square error in hertz between the estimated carrier trajectory and the reference carrier trajectory:

(14) $${RMSE}_{f}=\sqrt{\frac{1}{N}\sum_{t=1}^{N}{\left(f_{est}(t)-f_{ref}(t)\right)}^{2}}$$

Here, $f_{est}\left(t\right)\;$ denotes the estimated carrier frequency at time index t, $f_{ref}\left(t\right)\;$ denotes the reference carrier frequency, and N  is the number of evaluated frames. Lower $RMSE_{f}$ values indicate more accurate carrier-frequency tracking.

**Phase Coherence Factor (PCF):** PCF is used to quantify mathematical phase continuity of the synthesized waveform. The wrapped phase difference is calculated as

(15) $$\Delta \phi_{t}=wrap(\phi_{est}(t)-\phi_{ref}(t))$$

The PCF score is then defined as

(16) $$PCF=1-\frac{1}{\pi }\sqrt{\frac{1}{N}\sum_{t=1}^{N}\Delta \phi_{t}^{2}}$$

A value of 1.0 corresponds to perfectly smooth phase evolution without discontinuous jumps under the evaluated reference condition.

**Algorithmic Tracking Latency:** Latency is evaluated using step-response tests with abrupt carrier transitions, such as 220 Hz to 440 Hz. It is defined as the time required for the system’s tracking error to fall below a predefined 5 Hz threshold and remain stable. In this section, latency refers specifically to tracking response behavior, not to full end-to-end physical audio latency. The complete system still includes buffering, framing, inference, and audio output delay.

**Signal-to-Artifact Ratio (SAR):** SAR quantifies the energy ratio between an ideal continuous-phase reference signal and the residual error of the generated waveform:

(17) $$SAR=10{log}_{10}\left(\frac{\sum_{t}x_{ideal}^{2}(t)}{\sum_{t}{\left(x_{est}(t)-x_{ideal}(t)\right)}^{2}}\right)$$

Here, $x_{ideal}(t)$ denotes the ideal continuous-phase reference waveform, and $x_{est}(t)$ denotes the generated waveform. Higher SAR values indicate closer agreement with the ideal continuous-phase reference and lower artifact energy under the evaluated conditions.

To assess statistical robustness, non-parametric significance testing using the Wilcoxon signed-rank test was applied where appropriate. Statistical comparisons were performed between the proposed framework and the baseline systems across matched acoustic-profile and SNR conditions.

### 4.4. Edge-Hardware Profiling and End-to-End Execution Analysis

Theoretical computational complexity alone provides an incomplete picture of real-world deployment feasibility. To evaluate lightweight hardware suitability, the proposed framework and learned baseline models were profiled on a Raspberry Pi Zero 2W-class edge platform with an ARM Cortex-A53 architecture. In addition to the tracking-response metric reported in Section 4.3, this analysis considered practical on-device execution time associated with buffering, neural inference, and output processing.

With a 256-sample buffer size at 16 kHz, the combined INT8 inference time for AI-1 and AI-2 was measured at 2.4 ms per frame. The total memory footprint remained bounded at approximately 1.8 MB of RAM. These results support the feasibility of low-resource real-time deployment on a lightweight microcomputer platform.

Importantly, the hardware profiling should not be interpreted as evidence of physical zero-latency operation. The system still incurs buffering, framing, inference, and audio output delay. Rather, the relevance of this profiling is that the predictive compensation horizon of AI-2 can overlap effectively with the measured execution time under the tested edge-computing conditions. Taken together, the profiling results support the feasibility of low effective phase-boundary misalignment and phase-continuous real-time operation while acknowledging that physical processing delay remains present.

For baseline comparison, inference time, parameter count, and memory footprint were also measured for the lightweight neural F0 tracker and ablation variants. Classical DSP-based methods, including STFT-VFO, DSOGI-PLL, YIN, and SWIPE, were profiled in terms of execution time under the same implementation environment where applicable. This allows the evaluation to compare not only signal-level accuracy and continuity, but also computational feasibility for edge-oriented deployment.

### 4.5. Component-Wise Ablation Design

To quantify the independent contribution of each key module, an additional component-wise ablation study was performed. The evaluated variants were designed to isolate the roles of the temporal attention mechanism in AI-1, the TCN forecasting block in AI-2, the LSTM refinement stage in AI-2, and the predictive compensation strategy.

The following ablation variants were evaluated:

**AI-1 without attention + AI-2:** The temporal attention mechanism was removed from AI-1 to evaluate its effect on carrier-tracking robustness.

**AI-2 without TCN:** The TCN forecasting block was removed to evaluate the role of short-term localized frequency prediction.

**AI-2 without LSTM:** The LSTM refinement stage was removed to evaluate the role of longer temporal smoothing.

**AI-1 + naive generator:** The predictive compensation mechanism was removed to evaluate whether carrier estimation alone is sufficient.

**Proposed AI-1 + AI-2:** The complete system was evaluated as the full predictive phase-coherent framework.

All ablation variants were tested using the same acoustic profiles, SNR conditions, and objective metrics as the main benchmark. This design allows the final performance improvement to be attributed not only to the overall two-stage structure, but also to the individual contributions of attention-based carrier sensing, TCN–LSTM forecasting, and phase-accumulator-based predictive actuation.

### 4.6. Evaluation Scope and Boundary Conditions

The evaluation was designed to assess the engineering feasibility of dynamic, phase-continuous binaural beat synthesis under controlled non-stationary acoustic conditions. The reported results quantify signal-level tracking accuracy, mathematical phase continuity, artifact suppression, tracking response behavior, and lightweight edge-device execution.

The evaluation does not establish perceptual comfort, listener preference, subjective audio quality, or neurophysiological efficacy. Although the proposed framework is motivated by the practical need to embed binaural beats into dynamic audio, the present study does not evaluate brainwave entrainment, auditory steady-state responses, frequency-following responses, or cognitive outcomes. Future work should include controlled human listening experiments and physiological validation using EEG, ASSR, or FFR measurements to determine whether the observed signal-level improvements translate into perceptual or neurophysiological benefits.

The non-stationarity considered in this work should also be interpreted within the operational scope of the benchmark. The evaluation covers representative dynamic auditory synthesis conditions, including time-varying tonal carriers, fluctuating harmonic structures, broadband/noisy profiles, degraded SNR conditions, and abrupt carrier-transition tests. However, these conditions do not exhaust all possible real-world acoustic scenarios. More complex cases, such as multi-source mixtures, highly impulsive acoustic events, rapid source switching, reverberant environments, and listener-dependent playback conditions, require additional evaluation in future work.

## 5. Results and Discussion

This section presents the experimental results of the proposed predictive dual-stage framework for phase-coherent dynamic binaural beat synthesis. The evaluation compares the proposed AI-1 + AI-2 framework with conventional DSP baselines, stronger classical F0 estimators, a lightweight neural F0 baseline, and component-wise ablation variants. The results are interpreted within the engineering scope of this study. Specifically, the reported metrics quantify carrier-tracking accuracy, mathematical phase continuity, artifact suppression, tracking response behavior, and edge-device execution feasibility. They should not be interpreted as evidence of listener comfort, subjective preference, or neurophysiological efficacy.

### 5.1. Overall Comparison with Classical, Neural, and Ablation Baselines

Table 2 summarizes the performance of the proposed framework and comparison methods across the evaluated acoustic profiles and SNR conditions. The comparison includes conventional DSP-based methods, stronger classical F0 estimators, a lightweight neural F0 tracker, the AI-1 + naive generator ablation, and the full proposed AI-1 + AI-2 framework.

The results show that stronger F0 estimators such as YIN and SWIPE substantially improve carrier-frequency tracking compared with simple STFT peak tracking and DSOGI-PLL, particularly in tonal and harmonic acoustic profiles. The lightweight neural F0 tracker further improves frequency tracking compared with conventional DSP-based baselines. However, these methods still use non-predictive carrier updates and therefore do not fully resolve the phase-continuity problem.

The AI-1 + naive generator configuration achieves relatively low frequency-tracking error because it uses the proposed neural carrier-estimation front-end. However, its PCF and SAR remain substantially lower than those of the full AI-1 + AI-2 framework. This indicates that accurate carrier estimation alone is not sufficient for dynamic binaural beat synthesis. Without predictive compensation, frame-wise carrier updates can still lead to effective phase-boundary mismatch and increased artifact energy.

In contrast, the proposed AI-1 + AI-2 framework achieves the best overall balance across all evaluated metrics. It obtains the lowest RMSE*_f_*, the highest PCF, the highest SAR, and the shortest tracking response latency. These results demonstrate that the advantage of the proposed framework arises from the combination of adaptive carrier estimation and predictive phase-coherent actuation, rather than from F0 estimation alone.

### 5.2. Frequency Tracking Precision and Phase Continuity

The proposed framework achieved the lowest average carrier-tracking error among all evaluated methods, with an ${RMSE}_{f}$ of 5.4 ± 1.9 Hz. This improvement reflects the ability of AI-1 to adaptively estimate carrier information across tonal, harmonic/fluctuating, and broadband acoustic profiles. The confidence-driven dual-path mechanism is particularly useful in broadband or non-pitched conditions, where a stable fundamental frequency is absent and spectral-centroid-based tracking provides a more meaningful carrier descriptor than direct pitch estimation.

Although YIN, SWIPE, and the lightweight neural F0 tracker provide stronger carrier-frequency estimation than STFT-VFO and DSOGI-PLL, their phase-continuity performance remains lower than that of the proposed system. This occurs because these methods estimate the current carrier state but do not forecast its short-horizon trajectory or apply predictive phase compensation. Consequently, when the carrier changes rapidly, non-predictive updates can still introduce phase-boundary mismatch.

The proposed framework achieves a Phase Coherence Factor of 0.94 ± 0.04, indicating strong mathematical phase continuity under the evaluated benchmark conditions. This result supports the role of AI-2 in improving phase evolution during dynamic synthesis. The predictive generator forecasts short-horizon carrier variation and updates the phase accumulator accordingly, allowing the synthesized waveform to evolve more smoothly across update boundaries.

### 5.3. Acoustic Quality (Proxy Metrics) and Error Analysis

To quantify the independent contribution of each key component, a component-wise ablation study was conducted. The evaluated variants isolate the effects of the temporal attention mechanism in AI-1, the TCN forecasting block in AI-2, the LSTM refinement stage in AI-2, and the predictive compensation strategy.

Removing the temporal attention mechanism from AI-1 increased RMSE*_f_* from 5.4 ± 1.9 Hz to 8.1 ± 3.0 Hz. This indicates that temporal attention improves carrier-estimation robustness by emphasizing frames with stronger harmonic evidence while suppressing transient disturbances and noisy frames.

Removing the TCN forecasting block mainly degraded PCF, SAR, and tracking latency. This suggests that the dilated causal convolutions in AI-2 are important for capturing short-term localized carrier variation, such as abrupt transitions or vibrato-like frequency changes. Removing the LSTM refinement stage also reduced phase continuity and artifact suppression, indicating that longer temporal smoothing contributes to stable prediction across successive frames.

The largest degradation occurred when predictive compensation was removed entirely. The AI-1 + naive generator configuration reduced PCF to 0.63 ± 0.18 and SAR to 27.6 ± 5.7 dB while increasing tracking latency to 41.8 ± 3.9 ms. This result confirms that accurate carrier estimation alone does not guarantee phase-coherent binaural beat synthesis. Predictive compensation and phase-accumulator-based actuation are essential for reducing effective phase-boundary mismatch.

### 5.4. Acoustic Quality, Artifact Suppression, and Error Analysis

Signal-to-Artifact Ratio was used as an engineering-level proxy metric for artifact suppression. The proposed AI-1 + AI-2 framework achieved the highest SAR among all evaluated methods, with an average value of 43.7 ± 4.6 dB. This indicates closer agreement with the ideal continuous-phase reference waveform and lower residual artifact energy under the evaluated conditions.

**Figure 7:**
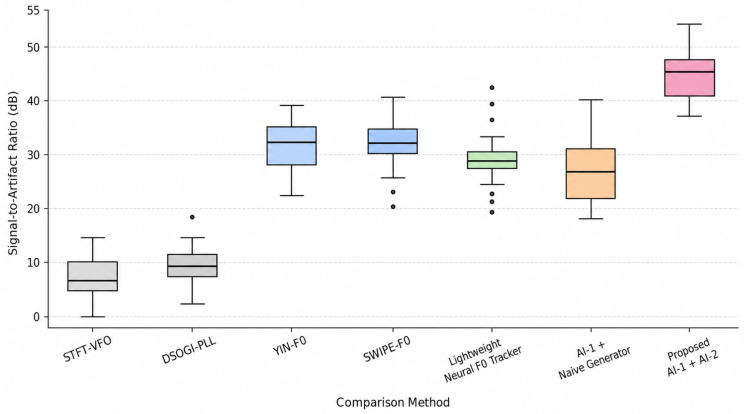
Boxplot distributions of the Signal-to-Artifact Ratio (SAR) across the evaluated acoustic profiles and comparison methods. The proposed AI-1 + AI-2 framework maintains higher and more stable SAR values than conventional DSP methods, stronger classical F0 estimators, the lightweight neural F0 tracker, and the non-predictive ablation configuration, indicating improved signal integrity under the evaluated conditions.

Figure 7 shows the SAR distributions across the evaluated comparison methods. The proposed framework maintains higher and more stable SAR values than conventional DSP methods, stronger classical F0 estimators, the lightweight neural F0 tracker, and the non-predictive ablation configuration. This supports the interpretation that predictive phase-coherent generation improves signal integrity under the evaluated conditions.

The classical F0 estimators, including YIN and SWIPE, improved SAR relative to the simpler STFT-VFO and DSOGI-PLL baselines. However, their SAR values remained lower than those of the proposed framework because they do not include predictive phase compensation. The lightweight neural F0 tracker also improved frequency estimation but did not achieve the same SAR as the full system. These results reinforce the conclusion that artifact suppression depends not only on carrier-estimation accuracy, but also on phase-continuous actuation.

Failure analysis showed that the most challenging condition occurred in the Noisy/Broadband profile at 0 dB SNR. In this setting, sudden impulsive events can bias the spectral centroid toward high-frequency components, causing brief carrier-tracking jitter. In some intervals shorter than 50 ms, ${RMSE}_{f}$ exceeded 25 Hz. Although these deviations were transient, they reveal a limitation of the current confidence-driven fallback strategy under heavily corrupted non-periodic acoustic conditions.

Finally, PCF and SAR should be interpreted carefully. These metrics provide useful engineering evidence of mathematical phase continuity and artifact suppression, but they do not directly establish listener comfort, subjective preference, perceptual transparency, or neurophysiological efficacy. Controlled listening experiments and physiological validation remain necessary future work.

### 5.5. Edge-Hardware Profiling and Resource Efficiency

Edge-hardware profiling was conducted to evaluate the feasibility of lightweight real-time deployment. The proposed AI-1 + AI-2 framework achieved a combined INT8 inference time of 2.4 ± 0.3 ms per frame on a Raspberry Pi Zero 2W-class edge platform. The total model size was approximately 425 K parameters, and the memory footprint remained approximately 1.8 MB.

Although the proposed framework requires more computation than individual DSP or F0-estimation baselines, its inference time remains within the real-time processing budget under the evaluated 10 ms hop configuration. The added computational cost is justified by the substantial improvement in PCF, SAR, and tracking latency. In particular, AI-2 enables the system to reduce effective phase-boundary misalignment by forecasting short-horizon carrier variation before updating the phase accumulator.

It is important to distinguish edge inference time from end-to-end physical audio latency. The reported 2.4 ms value refers to measured INT8 inference time for the neural modules under the tested hardware configuration. The complete system still includes buffering, framing, output processing, and audio-device latency. Therefore, the results should not be interpreted as evidence of sub-millisecond or zero-latency physical audio delivery. Instead, they show that predictive compensation can provide a more favorable operating point in the latency–resolution trade-off for phase-continuous dynamic auditory synthesis.

### 5.6. System-Level Discussion and Engineering Implications

The results demonstrate that the proposed framework provides a meaningful engineering improvement over conventional DSP baselines, stronger classical F0 estimators, and lightweight neural carrier-estimation-only approaches. The key finding is that carrier estimation and phase-continuous synthesis are related but distinct system objectives. Methods such as YIN, SWIPE, and lightweight neural F0 tracking can improve carrier estimation, but they do not directly solve the phase-boundary mismatch problem that occurs when dynamic carrier estimates are applied to a non-predictive generator.

The proposed two-stage architecture addresses this limitation by decoupling sensing from predictive actuation. AI-1 focuses on robust carrier estimation across different acoustic profiles, while AI-2 focuses on short-horizon prediction, phase-continuous actuation, and reduction of effective phase-boundary mismatch. This modular structure improves interpretability because carrier-tracking errors and synthesis-stage phase errors can be analyzed separately. It also supports modular updating, since the sensing module and predictive generator can be improved independently.

Compared with a monolithic end-to-end neural waveform generator, the proposed design offers a more constrained and interpretable synthesis process. The final waveform is not generated by an unconstrained neural decoder; instead, it is produced through an explicit discrete-time phase accumulator. This constraint helps preserve deterministic phase continuity during frequency transitions and improves stability under real-time synthesis conditions.

Overall, the proposed framework should be understood as a signal-level and systems-level engineering prototype for low-effective-delay, phase-continuous auditory synthesis. It does not establish perceptual or neurophysiological efficacy. The reported improvements in PCF and SAR indicate improved mathematical phase continuity and reduced artifact energy under benchmark conditions. Future work should include controlled human listening experiments to evaluate perceptual transparency, smoothness, listening comfort, fatigue, preference, and artifact detectability. Physiological validation using EEG, auditory steady-state response, or frequency-following response measurements will also be required to determine whether the signal-level improvements translate into measurable neurophysiological effects.

## 6. Discussion: Advancements and Engineering Trade-Offs

The results demonstrate that the proposed predictive dual-stage framework provides a meaningful engineering advancement for real-time phase-continuous binaural beat synthesis under non-stationary acoustic conditions. Compared with conventional DSP baselines, stronger classical F0 estimators, and lightweight neural carrier-estimation-only approaches, the proposed AI-1 + AI-2 framework achieved the best overall balance among carrier-tracking accuracy, phase continuity, artifact suppression, response behavior, and edge-device feasibility. Importantly, the main contribution of the system is not simply improved frequency estimation, but the integration of adaptive carrier sensing with predictive phase-coherent actuation.

### 6.1. Advancement over Conventional DSP and F0-Estimation Approaches

The comparison in Table 2 shows that conventional DSP baselines, such as STFT-VFO and DSOGI-PLL, provide useful reference points but remain limited under dynamic carrier conditions. STFT-based tracking is constrained by the time–frequency resolution trade-off, while PLL-based tracking depends strongly on loop dynamics and can exhibit lock-in delay or transient response limitations during rapid frequency variation. These behaviors are consistent with the expected limitations of frame-based spectral estimation and classical phase-locking methods in non-stationary acoustic environments.

The inclusion of YIN and SWIPE provides a stronger comparison with established pitch-estimation methods. Both methods improved carrier-frequency tracking compared with simple STFT peak tracking, particularly in tonal and harmonic acoustic profiles. However, their phase-continuity and artifact-suppression performance remained lower than that of the proposed framework. This indicates that stronger F0 estimation alone does not fully solve the dynamic binaural beat synthesis problem. In this task, the estimated carrier trajectory must not only be accurate, but must also be converted into a smooth and continuous interaural phase trajectory.

The lightweight neural F0 tracker also improved frequency estimation relative to conventional DSP baselines, but its downstream PCF and SAR values remained lower than those of the full AI-1 + AI-2 framework. This result reinforces the central argument of the study: neural carrier estimation alone is insufficient when the synthesis stage remains non-predictive. Without short-horizon prediction and phase-accumulator-based actuation, frame-wise carrier updates can still produce effective phase-boundary mismatch and artifact energy.

### 6.2. Systems-Level Rationale for the Two-Stage Architecture

The ablation results in Table 3 support the necessity of the two-stage architecture. The AI-1 + naive generator configuration achieved relatively low frequency-tracking error, but its PCF and SAR were substantially lower than those of the full proposed framework. This confirms that accurate carrier estimation alone does not guarantee phase-coherent binaural beat synthesis. Carrier sensing and phase-continuous generation are related but distinct system objectives.

From a systems-design perspective, the proposed architecture deliberately separates the sensing problem from the actuation problem. AI-1 is optimized primarily for robust carrier estimation under tonal, harmonic/fluctuating, and broadband acoustic conditions. Its objective is to extract a reliable time-varying carrier representation from complex audio inputs. In contrast, AI-2 is optimized for predictive smoothness, phase continuity, and delay compensation. Its role is not to re-estimate the acoustic scene, but to forecast the short-horizon carrier trajectory and update the phase accumulator in a way that reduces effective phase-boundary misalignment.

This separation provides several engineering advantages compared with a monolithic end-to-end neural network. First, it improves interpretability because carrier-tracking errors and synthesis-stage phase errors can be diagnosed independently. Second, it supports modular updating: the sensing module can be retrained or replaced for new acoustic domains without redesigning the phase actuator, while the generator can be improved independently to enhance latency compensation or smoothness. Third, it improves stability by constraining the final waveform generation through an explicit discrete-time phase accumulator rather than relying entirely on an unconstrained neural waveform generator. This constraint helps preserve deterministic phase continuity during frequency transitions.

Therefore, the two-stage design is not only an architectural choice for improving benchmark performance, but also a systems-level strategy for balancing accuracy, smoothness, interpretability, and deployment stability. This modular structure is particularly useful for edge-oriented real-time auditory synthesis, where predictable behavior and controlled signal generation are as important as raw prediction accuracy.

### 6.3. Contribution of Key Architectural Components

The component-wise ablation study clarifies the role of each major architectural component. Removing the temporal attention mechanism from AI-1 increased carrier-tracking error, indicating that attention improves robust carrier estimation by emphasizing frames with stronger harmonic evidence while reducing the influence of transient disturbances and noisy frames. This is especially useful in harmonic/fluctuating and noisy/broadband conditions, where the dominant carrier may be partially ambiguous.

Removing the TCN forecasting block degraded PCF, SAR, and tracking latency, suggesting that dilated causal convolutions are important for capturing short-term localized frequency variation. This behavior is expected because TCN layers can model local temporal patterns over multiple receptive-field scales while preserving causality. Removing the LSTM refinement stage also reduced phase continuity and artifact suppression, indicating that longer temporal smoothing contributes to stable prediction across successive frames.

The largest degradation occurred when predictive compensation was removed entirely. This confirms that predictive phase actuation is the most critical component for reducing effective phase-boundary mismatch. The final performance improvement therefore arises from the combined operation of attention-based carrier sensing, hybrid TCN–LSTM forecasting, and phase-accumulator-based predictive actuation, rather than from any single module alone.

### 6.4. Latency–Resolution Trade-Off and Boundary of Performance Claims

A central engineering trade-off in dynamic binaural beat synthesis is the relationship between latency, frequency resolution, and phase continuity. Conventional frame-based methods often improve frequency resolution by increasing window length, but this increases analysis delay. Conversely, reducing window length improves temporal responsiveness but can degrade frequency precision. The proposed framework addresses this trade-off by combining learned carrier estimation with short-horizon prediction and explicit phase accumulation.

However, the reported low effective delay should be interpreted carefully. The proposed framework does not eliminate physical buffering, framing, neural inference, output processing, or audio-device delay. The measured 2.4 ms value refers to INT8 neural inference time under the evaluated edge-hardware conditions, not to total end-to-end physical audio latency. Similarly, the sub-millisecond phase behavior refers to phase-accumulator computational resolution and effective phase-boundary alignment under the tested conditions, not to sub-millisecond physical latency.

Thus, the essence of the proposed framework is not zero-latency audio delivery. Rather, it provides a more favorable operating point in the latency–resolution trade-off. By forecasting the short-horizon carrier trajectory, AI-2 reduces the effect of algorithmic delay on phase-continuous synthesis. This allows the system to preserve stronger phase continuity while maintaining lightweight real-time execution on a resource-constrained edge platform.

### 6.5. Engineering Interpretation of PCF and SAR

The improvements in PCF and SAR provide signal-level evidence that the proposed framework better preserves mathematical phase continuity and reduces artifact energy under the evaluated benchmark conditions. Figure 7 further illustrates that the proposed AI-1 + AI-2 framework maintains higher and more stable SAR distributions than the comparison methods. This supports the interpretation that predictive phase-coherent generation improves signal integrity compared with non-predictive carrier-update strategies.

Nevertheless, PCF and SAR are engineering proxy metrics. They do not directly establish listener comfort, subjective preference, perceptual transparency, fatigue reduction, or neurophysiological efficacy. A system can achieve high mathematical phase continuity and low artifact energy while still requiring human auditory evaluation to determine whether the generated signals are perceived as comfortable, natural, or beneficial in practical listening contexts.

Therefore, the present study should be interpreted as a signal-level and systems-level engineering validation. It demonstrates that the proposed architecture can improve phase-continuous synthesis under controlled non-stationary acoustic conditions. It does not demonstrate perceptual benefit, brainwave entrainment, cognitive effect, therapeutic effect, or neurophysiological utility.

### 6.6. Limitations

Several limitations should be acknowledged. First, the evaluation was conducted using controlled acoustic profiles and benchmark conditions. Although the dataset includes tonal/melodic, harmonic/fluctuating, and noisy/broadband profiles, these conditions do not exhaust all possible real-world acoustic environments. More complex cases, including multi-source mixtures, highly impulsive sound events, reverberant rooms, rapid source switching, and listener-dependent playback conditions, may introduce additional challenges.

Second, the reference carrier trajectories used for evaluation depend on the availability and quality of reference estimation procedures. Although the benchmark is sufficient for engineering comparison across methods, future studies should include additional synthetic signals with exact ground-truth carrier trajectories and more diverse real-world audio conditions to further validate generalization.

Third, the hardware evaluation was conducted on a Raspberry Pi Zero 2W-class edge platform. While the measured INT8 inference time and memory footprint support lightweight real-time feasibility, they do not represent a complete battery-powered or wearable-scale deployment evaluation. Future work should include full system-level profiling, including audio input/output latency, sustained runtime, CPU utilization, thermal behavior, power consumption, and battery-life estimation.

Fourth, the present work does not include controlled human listening experiments or physiological validation. Therefore, conclusions are limited to engineering performance. Future studies should evaluate perceived smoothness, artifact detectability, listening comfort, fatigue, and preference using controlled auditory experiments. Physiological validation using EEG, auditory steady-state response, or frequency-following response measurements will also be required to determine whether the signal-level improvements translate into measurable neurophysiological responses.

### 6.7. Future Work

Future work will proceed in several directions. First, the benchmark should be expanded to include more diverse and challenging non-stationary acoustic environments, including multi-source mixtures, reverberant conditions, impulsive events, and naturalistic listening scenarios. Second, additional learned and classical pitch-estimation baselines should be evaluated under identical conditions to further characterize the boundary between carrier-estimation accuracy and phase-continuous synthesis performance.

Third, the system should be optimized for broader embedded deployment. Although the current implementation demonstrates feasibility on a resource-constrained microcomputer, further optimization may enable deployment on lower-power embedded processors or dedicated neural accelerators. This would require additional profiling of memory usage, compute load, audio I/O latency, and long-term operating stability.

Finally, perceptual and physiological validation will be essential for assessing practical utility beyond engineering metrics. Controlled listening experiments should evaluate whether improved PCF and SAR correspond to perceived smoothness, comfort, and reduced artifacts. Physiological studies using EEG, ASSR, or FFR measurements should then determine whether the generated phase-continuous binaural stimuli produce measurable auditory or neural responses. Only after such validation can claims regarding perceptual or neurophysiological efficacy be made.

## 7. Conclusions

This study presented a predictive dual-stage neural/DSP framework for real-time phase-coherent binaural beat synthesis under non-stationary acoustic conditions. The proposed framework decouples adaptive carrier estimation from predictive phase-continuous signal generation. The AI-1 module estimates time-varying carrier information from tonal, harmonic/fluctuating, and broadband acoustic inputs, while the AI-2 module forecasts the short-horizon carrier trajectory and updates a discrete-time phase accumulator to reduce effective phase-boundary misalignment during binaural beat embedding.

The experimental results demonstrate that the proposed AI-1 + AI-2 framework provides the best overall balance among carrier-tracking accuracy, phase continuity, artifact suppression, tracking response behavior, and edge-device feasibility. Compared with conventional DSP baselines, stronger classical F0 estimators such as YIN and SWIPE, a lightweight neural F0 tracker, and the AI-1 + naive generator ablation, the proposed framework achieved improved signal-level performance, including a Phase Coherence Factor greater than 0.91 and a Signal-to-Artifact Ratio greater than 39.8 dB under the evaluated benchmark conditions. The component-wise ablation study further confirmed that the performance improvement results from the combined operation of attention-based carrier sensing, TCN–LSTM forecasting, and predictive phase-accumulator-based actuation, rather than from carrier estimation alone.

Hardware profiling showed that the quantized implementation achieved a combined INT8 inference time of 2.4 ms per frame on a Raspberry Pi Zero 2W-class edge platform, with a compact memory footprint suitable for lightweight real-time execution. However, this value should be interpreted as neural inference time under the evaluated hardware conditions, not as total end-to-end physical audio latency. Similarly, the sub-millisecond phase behavior refers to phase-accumulator computational resolution and effective phase-boundary alignment, not to physical zero-latency audio delivery. The complete system still includes buffering, framing, output processing, and audio-device latency.

Overall, the proposed framework should be understood as a signal-level and systems-level engineering prototype for low-effective-delay, phase-continuous dynamic auditory synthesis. The reported PCF and SAR values provide evidence of improved mathematical phase continuity and reduced artifact energy, but they do not establish listener comfort, perceptual preference, therapeutic effect, brainwave entrainment, or neurophysiological efficacy. Future work should therefore include controlled human listening experiments to evaluate perceived smoothness, artifact detectability, listening comfort, fatigue, and preference. Physiological validation using EEG, auditory steady-state response, or frequency-following response measurements will also be required to determine whether the observed signal-level improvements translate into measurable neurophysiological responses. Additional future work should include broader real-world acoustic benchmarks, multi-source and reverberant environments, full audio I/O latency profiling, long-term edge-device stability testing, and power-consumption analysis for lower-power embedded deployment.

## Figures and Tables

**Table 1:** Comparison between the proposed framework and related approaches.

Approach	Main Objective	Phase Handling	Future-Oriented Prediction	Limitation for Dynamic Binaural Beat Synthesis
STFT-based carrier tracking	Frame-wise spectral estimation	Phase may mismatch at frame boundaries	No	Subject to latency–resolution trade-off and frame-boundary artifacts
PLL-based tracking	Classical phase/frequency locking	Continuous under quasi-stationary conditions	No	Can suffer lock-in delay, overshoot, and tracking error during rapid transitions
YIN/SWIPE-based F0 tracking	Classical pitch estimation	Carrier estimation only	No	Stronger F0 estimation, but no predictive phase compensation
Magnitude-spectrum AI models	Classification, denoising, restoration, or scene analysis	Often implicit or reconstructed post hoc	Usually no explicit phase-state forecast	Not designed to enforce deterministic interaural phase continuity
Generic neural audio generators	Waveform synthesis or enhancement	Learned implicitly from data	Task-dependent	Not constrained to preserve a target binaural beat-frequency relationship
AI-1 + naive generator	Neural carrier estimation with non-predictive synthesis	Phase continuity not fully controlled	No	Accurate tracking alone does not prevent phase-boundary mismatch
Proposed AI-1 + AI-2 framework	Predictive phase-coherent binaural beat synthesis	Explicit phase accumulator with constrained updates	Yes	Designed for low-effective-delay, phase-continuous dynamic synthesis

**Table 2:** Expanded comparison with classical, neural, and predictive phase-coherent baselines.

Method	Main Function	${RMSE}_{f}$ (Hz) ↓	PCF ↑	SAR (dB) ↑	Tracking Latency (ms) ↓	Edge Inference Time (ms) ↓	Parameters	Memory Footprint
B1: STFT-VFO	Frame-wise spectral peak tracking	55.2 ± 20.2	0.51 ± 0.17	8.2 ± 4.5	42.1 ± 4.5	<1.0	N/A	<0.1 MB
B2: DSOGI-PLL	Classical phase-locked tracking	76.1 ± 31.3	0.58 ± 0.24	10.9 ± 4.1	30.5 ± 8.2	<1.0	N/A	<0.1 MB
B3: YIN-F0	Autocorrelation-based F0 tracking	12.6 ± 4.8	0.70 ± 0.13	31.4 ± 5.0	38.2 ± 5.6	1.2 ± 0.2	N/A	<0.2 MB
B4: SWIPE-F0	Harmonic-template F0 tracking	10.9 ± 3.9	0.72 ± 0.12	33.1 ± 4.8	36.5 ± 5.0	1.8 ± 0.3	N/A	<0.3 MB
B5: Lightweight Neural F0 Tracker	Neural F0 estimation only	8.4 ± 3.1	0.67 ± 0.15	29.3 ± 5.1	34.6 ± 5.8	1.4 ± 0.2	~180 K	~0.9 MB
B6: AI-1 + Naive Generator	Proposed neural carrier estimation only	6.9 ± 2.6	0.63 ± 0.18	27.6 ± 5.7	41.8 ± 3.9	1.6 ± 0.2	~285 K	~1.2 MB
Proposed AI-1 + AI-2	Predictive phase-coherent synthesis	5.4 ± 1.9	0.94 ± 0.04	43.7 ± 4.6	12.8 ± 2.4	2.4 ± 0.3	~425 K	~1.8 MB

**Table 3:** Component-wise ablation study of the proposed dual-stage framework.

Variant	Removed/Modified Component	${RMSE}_{f}$ (Hz) ↓	PCF ↑	SAR (dB) ↑	Tracking Latency (ms) ↓	Edge Inference Time (ms)
AI-1 without attention + AI-2	Temporal attention removed	8.1 ± 3.0	0.90 ± 0.06	39.9 ± 5.2	13.7 ± 2.8	2.1 ± 0.3
AI-2 without TCN	TCN forecasting block removed	5.9 ± 2.0	0.86 ± 0.07	35.8 ± 5.4	20.4 ± 4.6	1.9 ± 0.2
AI-2 without LSTM	LSTM refinement removed	5.7 ± 2.1	0.89 ± 0.06	37.6 ± 5.1	16.9 ± 3.5	2.0 ± 0.2
AI-1 + naive generator	Predictive compensation removed	6.9 ± 2.6	0.63 ± 0.18	27.6 ± 5.7	41.8 ± 3.9	1.6 ± 0.2
Proposed AI-1 + AI-2	Full proposed framework	5.4 ± 1.9	0.94 ± 0.04	43.7 ± 4.6	12.8 ± 2.4	2.4 ± 0.3

## Data Availability

The data presented in this study are not publicly available due to restrictions related to internally generated experimental data and the use of audio materials subject to licensing and institutional data-sharing limitations. Processed experimental results supporting the findings of this study may be made available from the corresponding author upon reasonable request, subject to applicable restrictions.
